# Early maternal loss affects social integration of chimpanzees throughout their lifetime

**DOI:** 10.1038/srep16439

**Published:** 2015-11-10

**Authors:** Elfriede Kalcher-Sommersguter, Signe Preuschoft, Cornelia Franz-Schaider, Charlotte K. Hemelrijk, Karl Crailsheim, Jorg J. M. Massen

**Affiliations:** 1 Institute of Zoology, University of Graz, Universitaetsplatz 2, 8010 Graz, Austria; 2 Competence Center Apes, Four Paws, Linke Wienzeile 236, 1150 Vienna, Austria; 3 Groningen Institute for Evolutionary Life Sciences, University of Groningen, Nijenborgh 7, 9747 AG Groningen, The Netherlands; 4 Department of Cognitive Biology, University of Vienna, Althanstrasse 14, 1090 Vienna, Austria; 5 Department of Animal Ecology, Utrecht University, Padualaan 8, 3584 CH Utrecht, The Netherlands

## Abstract

The long-term effects of early adverse experiences on later psychosocial functioning are well described in humans, but sparsely documented for chimpanzees. In our earlier studies, we investigated the effects of maternal and social deprivation on three groups of ex-laboratory chimpanzees who experienced either an early or later onset of long-term deprivation. Here we expand our research by adding data on subjects that came from two stable zoo groups. The groups comprised of early maternally deprived wild-caught chimpanzees and non-deprived zoo-born chimpanzees. We found that compared to zoo chimpanzees, ex-laboratory chimpanzees were more restricted regarding their association partners in the newly formed groups, but not during their second year of group-life, indicating that social stability has an important influence on the toleration of association partners close-by. Social grooming activity, however, was impaired in early long-term deprived ex-laboratory chimpanzees as well as in early maternally deprived zoo chimpanzees compared to non-deprived zoo chimpanzees. Thus, we conclude that early maternal loss has lifelong effects on the social integration of chimpanzees which becomes evident in their grooming networks. Although the retrospective nature of our study prevents a clear causal explanation, our results are of importance for understanding the development of social competence in chimpanzees.

In highly social species where social partners represent resources which can have decisive effects on an individual’s fitness and wellbeing, social competence requires the capability to establish and maintain positive social relationships^1^,^2^,^3^,^4^. In humans, this capability to form bonds is a key part in defining social competence and is based on various socio-emotional and cognitive skills of the relationship partners^5^. These skills start to develop in infancy and continue to develop with age^6^. A sensitive and supportive care-giving environment resulting in a secure infant-caregiver bond, is thought to provide the basis for successful social adaptation by stimulating the development of socio-emotional skills, and is crucial for normal social development^7^,^8^. The repercussions of lacking this supportive care-giving environment are well documented^9^,^10^. The development of maladaptive disorganized attachments to caregivers and attachment disorders, i.e., the lack of selective attachments, are widespread among infants reared institutionally and/or experiencing severe neglect^11^,^12^,^13^,^14^. Likewise, early traumatic life events can cause socio-emotional problems in human toddlers^15^ and may account for the development of psychopathologies lasting into adulthood^16^.

Experimental studies on nonhuman primates revealed that the absence of a caregiver, i.e., the mother, can have profound and long-lasting effects on social competence of macaque infants’ too^17^,^18^,^19^, and that multiple transient separations of dependent infants from their mothers have long-term consequences^20^,^21^. This indicates that, similar to humans, social skill acquisition in nonhuman primate babies is normally built on the first social experiences with their caregiver. In chimpanzee infants, these first experiences arise primarily from their relationship with the mother, who provides care and contact during the first years of life^22^. Free-living chimpanzees live in highly complex societies with multi-dimensional and context-specific social relationships (e.g. ref. 23). The development of these social relationships is based on an infant-mother bond and subsequent social experience with ever-wider ranges of interaction partners. Therefore, it is not surprising that the negative effects of being orphaned are also evident in apes and include impaired acquisition of social skills^24^, diminished socio-emotional competence^25^, as well as vulnerability to psychopathologies^26^. Further, the rearing of chimpanzee infants in a laboratory has a negative impact on social cognition^27^. This is, however, ameliorated when chimpanzees are raised under responsive care conditions^27^ and in enculturated environments^28^,^29^. Moreover, the prevalence of disorganized attachments among severely deprived chimpanzee infants to human caregivers^30^ is comparable to that of institution-reared human children^12^,^13^,^14^. The effects of severe deprivation, including the rearing of new-born chimpanzees in total social isolation are well investigated (e.g. ref. 31, 32, 33)but only recently it has been shown that the negative effects of severe and long-term deprivation persist into adulthood in ex-laboratory chimpanzees, even after keeping conditions were enriched^34^,^35^,^36^,^37^. However, so far the documentation of long-term effects of early maternal loss derived from only one population of ex-laboratory chimpanzees.

Zoos worldwide hold founder chimpanzees who have been captured in the wild, before the CITES regulations were endorsed. These founder individuals experienced most likely early trauma in the form of maternal deprivation, preceded by witnessing the killing of their mother during capture^38^ and the subsequent dramatic change in living conditions from freedom to captivity. In contrast to the ex-laboratory chimpanzees, these founder orphans enjoyed responsive care by human surrogate mothers and/or peer rearing followed by a life in a social environment with several interaction partners of different demographic classes. We investigated retrospectively the impact of early maternal loss on later social integration by comparing these maternally deprived zoo chimpanzees with non-deprived zoo chimpanzees, and with ex-laboratory chimpanzees that were severely deprived on the long-term. We compared two stable social groups of zoo chimpanzees (Arnhem and Amersfoort, The Netherlands) comprising (1) zoo-born non-deprived individuals (ND) and (2) wild-caught early maternally deprived chimpanzees (EMD) with three social groups of ex-laboratory chimpanzees that all were caught in the wild, kept in the laboratory and then re-socialised in groups with different compositions. The ex-laboratory chimpanzees were either (3) early long-term deprived (ELD) or (4) late long-term deprived (LLD). Early maternally deprived zoo chimpanzees (EMD) were captured from the wild at a mean age of 1.6 years and were peer reared. The early and late long-term deprived ex-laboratory chimpanzees were housed solitarily for decades. The early deprived ones (ELD) were socially deprived at a mean age of 1.2 years, and the late deprived ones (LLD) at a mean age of 4.6 years (for more information see Methods and Supplementary Table 1). To investigate whether the ex-laboratory chimpanzees could recover from long-term deprivation we also included observations during their second year of group living.

The differences in social behaviour of early and late long-term deprived ex-laboratory chimpanzees are most visible in differential tolerance to passive close proximity and in grooming. These differences are attributable to the age at onset of social deprivation^35^,^36^,^37^. Tolerance of proximity and grooming are known to be important aspects of social competence in chimpanzees and to be dependent on the supportive environment as provided by the mother (close proximity^22^,^39^,^40^; grooming^22^,^41^). In this study, we used the same variables, but in a different operationalization, which captures the degree of integration into the subject’s social group. Social integration is an important aspect of social competence because it reflects an individual’s ability to socialise with different partners and thus the degree to which it is dependent on particular social partners.

We investigated these effects using Social Network Analysis, i.e., we calculated individual network measures, and ran Generalized Linear Mixed Models (GLMM) to compare network measures among individuals with different deprivation backgrounds, while controlling for sex, age and group size.

Based on the clear-cut differences we found previously between early and late long-term deprived ex-laboratory chimpanzees’ social competence^36^,^37^, we were interested in (1) how their social network measures would differ from non-deprived zoo chimpanzees and (2) whether the effects of early maternal deprivation are still evident in wild-caught orphaned chimpanzees after living in stable social groups for decades.

In this study we show that the toleration of conspecifics close-by seems to be affected by social stability as re-socialised ex-laboratory chimpanzees were more restricted regarding their association partners during their first year of group-life than were zoo chimpanzees that were living in stable social groups for years. Further evidence comes from the fact that ex-laboratory chimpanzees partially recovered during their second year of group-life. Social grooming, in contrast, was diminished or even lacking in chimpanzees deprived in early infancy compared to non-deprived chimpanzees. In other words, early maternally deprived zoo chimpanzees and early long-term deprived ex-laboratory chimpanzees both differed significantly from non-deprived mother reared zoo chimpanzees. Thus, it seems that maternal loss in early infancy affects the social grooming competence of chimpanzees in the long run.

## Results

For both dependent variables, toleration of passive close proximity and grooming, we calculated two different individual social network measures, the vertex strength centrality and the deviation from edge weight disparity, and compared these measures among the different deprivation classes, i.e., among early long-term deprived (ELD) as well as late long-term deprived (LLD) ex-laboratory chimpanzees and early maternally deprived (EMD) as well as non-deprived (ND) zoo chimpanzees. The *vertex strength centrality* reflects the standardized strength of association or grooming activity, expressed as the mean percent of scans an individual spent within an arm’s reach to or grooming of an individual group member. The *deviation from edge weight disparity* reflects how selectively a subject associates with or grooms its group mates, expressed as deviation from an equal distribution. Note that in both measures group size is taken into account (for details see Method section).

### Toleration of Passive Close Proximity (Fig. 1 for full networks)

**Figure 1:**
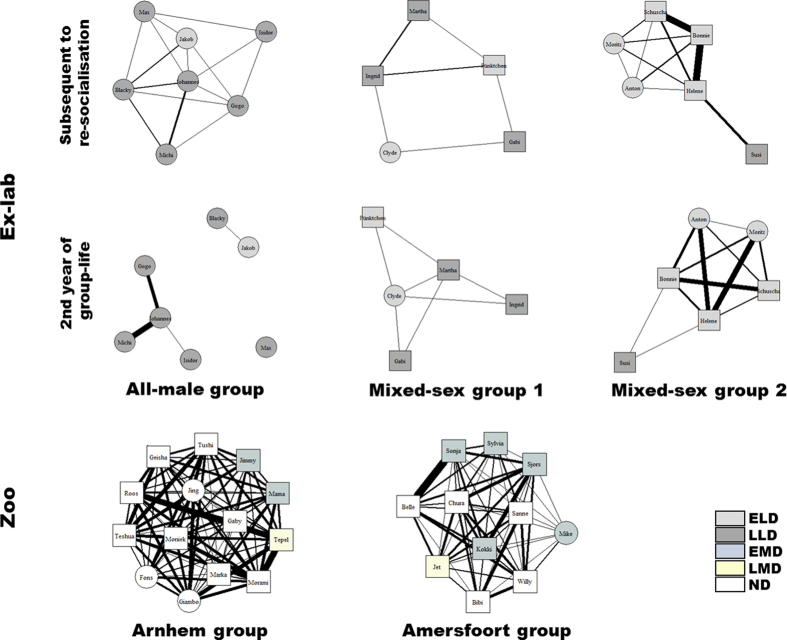
Close proximity networks of the five social groups. For ex-laboratory chimpanzees networks are shown subsequent to re-socialisation (2003) and during the second year of group-life (2005). Circles represent males, squares represent females, and undirected edges represent percent of scans a certain dyad spent within an arm’s reach. Abbr.: ELD = early long-term deprived, LLD = late long-term deprived, EMD = early maternally deprived, LMD = late maternally deprived (are not included in the analysis), ND = non-deprived.

It is apparent from Fig. 1 that the zoo groups formed much more tightly knit proximity networks than did two of the three ex-laboratory groups.

**Figure 2:**
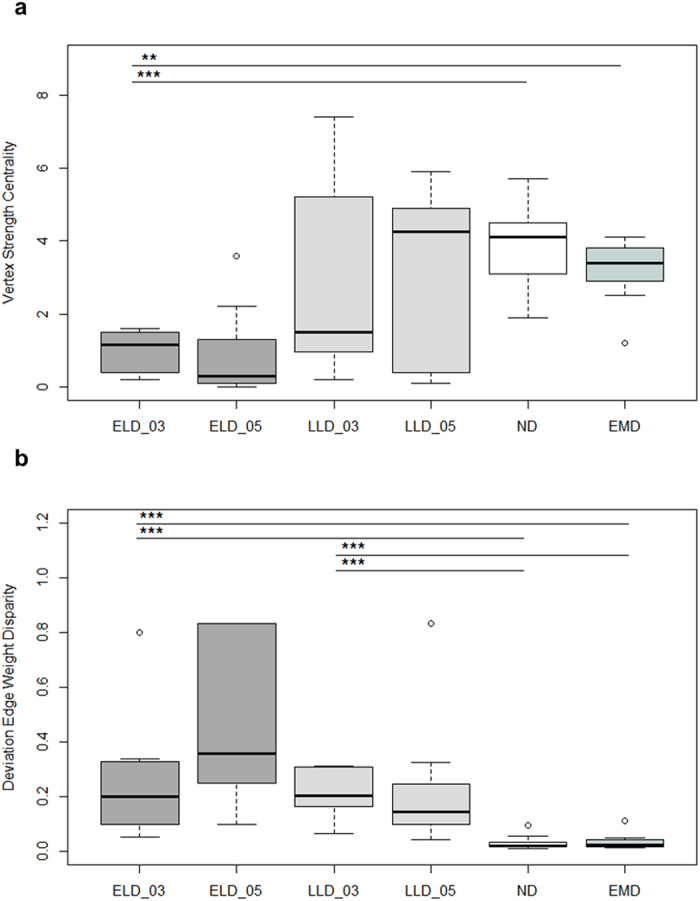
Close proximity network measures of the different deprivation classes. (**a**) Median vertex strength centrality, and (**b**) median deviation from edge weight disparity (interquartile range ± smallest and largest nonoutlier) of early long-term deprived individuals subsequent to re-socialisation (ELD_03), and during the second year of group life (ELD_05), late long-term deprived individuals subsequent to re-socialisation (LLD_03), and during the second year of group-life (LLD_05), early maternally deprived individuals (EMD), and non-deprived individuals (ND). **P < 0.01, ***P < 0.001 by post-hoc Mann-Whitney U test with Holm-Bonferroni correction.

Sex, deprivation class and the interaction of sex and deprivation class were significant predictors influencing *vertex strength centrality* (Table 1 & Supplementary Table 2). Females (*n* = 27) had a significantly higher vertex strength centrality than had males (*n* = 14; Mann-Whitney *U* = 65.0, *P* = 0.001). EMD (*n* = 7) and ND individuals (*n* = 16) had a significantly higher vertex strength centrality compared to ELD individuals (*n* = 10; ELD vs. EMD: Mann-Whitney *U* = 4.0, *P* = 0.001; ELD vs. ND: Mann-Whitney *U* = 0.0, *P* < 0.001; Fig. 2a). While males of the different deprivation classes did not differ significantly, most likely due to small sample sizes, we found EMD (*n* = 6) and ND females (*n* = 13) to have a significantly higher vertex strength centrality, i.e., were more strongly associated, than were ELD females (*n* = 4; ELD vs. EMD: Mann-Whitney *U* = 0.0, *P* = 0.010; ELD vs. ND: Mann-Whitney *U* = 0.0, *P* = 0.001).

Deprivation class was the only significant predictor influencing the *deviation from the edge weight disparity* (Table 1 & Supplementary Table 2). Both, EMD (*n* = 7) and ND individuals (*n* = 16) had a significantly lower deviation from edge weight disparity, i.e., were less selective in who they tolerated in an arm’s reach than the long-term deprived ELD (*n* = 10) and LLD individuals (*n* = 8; ELD vs. EMD: Mann-Whitney *U* = 3.0, *P* = 0.001; ELD vs. ND: *U* = 3.0, *P* < 0.001; LLD vs. EMD: *U* = 1.0, *P* = 0.001; LLD vs. ND: *U* = 1.0, *P* < 0.001; Fig. 2b).

#### Second year of group-life for ex-laboratory chimpanzees

For the ex-laboratory chimpanzees, we found marked differences with regard to integration in proximity networks between the period directly following upon re-socialisation and the second year of group-life. By the second year of group-life, none of the predictors significantly influenced the *vertex strength centrality*, or the *deviation from edge weight disparity* any longer (Table 1). This outcome is caused by a higher variability among both ELDs and LLDs (Fig. 2a,b) compared to the period subsequent to re-socialisation. While we found a decreased toleration of conspecifics close-by in most ELDs, most LLDs – but also some ELDs – ‘recovered’ within those two years of group-life (Fig. 1).

### Social Grooming Given (Fig. 3 for full networks)

**Figure 3:**
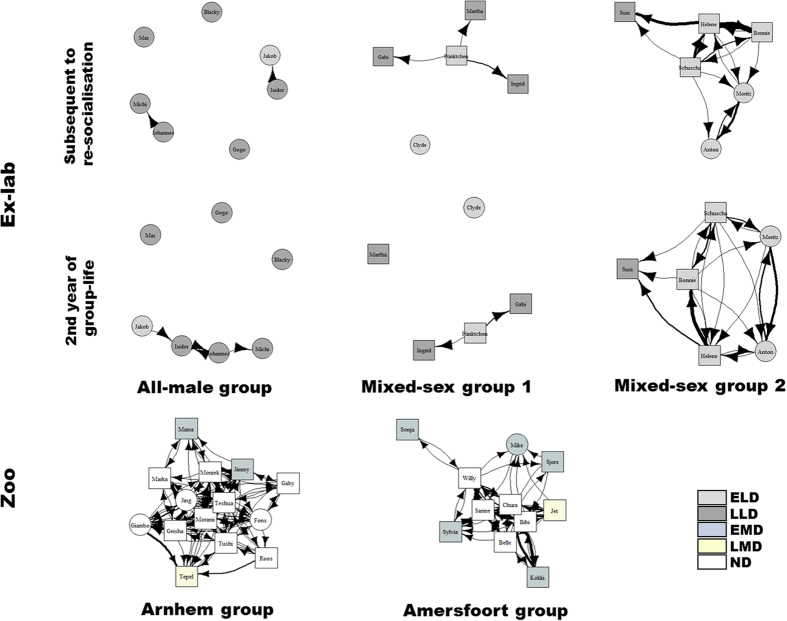
Social grooming networks of the five social groups. For ex-laboratory chimpanzees networks are shown subsequent to re-socialisation (2003) and during the second year of group-life (2005). Circles represent males, squares represent females, and edges are directed and represent percent of scans an individual spent on grooming the respective group member. Abbr.: ELD = early long-term deprived, LLD = late long-term deprived, EMD = early maternally deprived, LMD = late maternally deprived (are not included in the analysis), ND = non-deprived.

Figure 3 illustrates that the zoo groups and only one of the ex-lab groups formed grooming networks, and that even in these groups, ELDs and EMDs were more peripheral.

**Figure 4:**
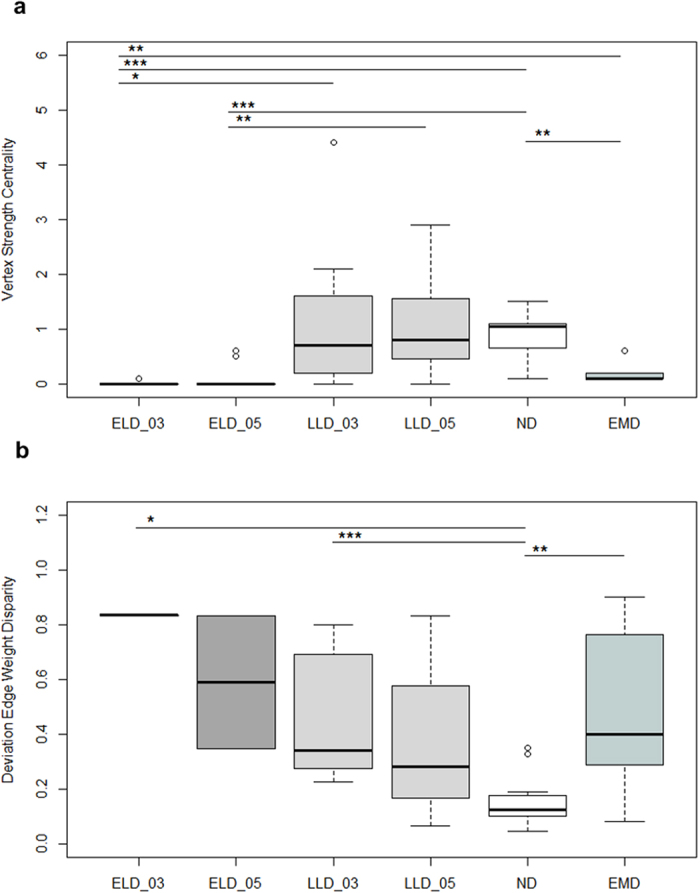
Grooming network measures of the different deprivation classes. (**a**) Median vertex strength centrality, and (**b**) median deviation from edge weight disparity (interquartile range ± smallest and largest nonoutlier) of early long-term deprived individuals subsequent to re-socialisation (ELD_03), and during the second year of group-life (ELD_05), late long-term deprived individuals subsequent to re-socialisation (LLD_03), and during the second year of group-life (LLD_05), early maternally deprived individuals (EMD), and non-deprived individuals (ND). *P < 0.05, **P < 0.01, ***P < 0.001 by post-hoc Mann-Whitney U test with Holm-Bonferroni correction.

Deprivation class was the only significant predictor influencing the *vertex strength centrality* (Table 2 & Supplementary Table 2). Compared to early long-term deprived (ELD) individuals (*n* = 10), LLD (*n* = 8), EMD (*n* = 7) and ND individuals (*n* = 16) had a significantly higher vertex strength centrality (ELD vs. LLD: Mann-Whitney *U* = 12.0, *P* = 0.014; ELD vs. EMD: *U* = 4.0, *P* = 0.003; ELD vs. ND: *U* = 1.0, *P* < 0.001; Fig. 4a). ELD chimpanzees had the lowest grooming activity. Importantly, however, also EMDs groomed significantly less than did NDs (lower vertex strength centrality: *U* = 8.5, *P* = 0.002, Fig. 4a). Post-hoc analysis revealed that neither males nor females differed significantly across different deprivation classes (most likely due to small sample sizes). Note that ELD females did not groom at all.

Sex, deprivation class and interaction of sex and deprivation class were significant predictors influencing the *deviation from the edge weight disparity* (Table 2 & Supplementary Table 2). However, post-hoc analysis revealed no significant difference between males (*n* = 8) and females (*n* = 23; Mann-Whitney *U* = 50.5, *P* = 0.064). ND individuals (*n* = 16) were significantly less selective regarding their grooming partners than were ELD (*n* = 2), LLD (*n* = 6) and EMD individuals (*n* = 7; ND vs. ELD: Mann-Whitney *U* = 0.0, *P* = 0.013; ND vs. LLD: *U* = 6.0, *P* = 0.001; ND vs. EMD: *U* = 18.0, *P* = 0.010; Fig. 4b). Again, neither males nor females differed significantly across different deprivation classes, most probably due to small sample sizes.

#### Second year of group-life for ex-laboratory chimpanzees

We found few differences with regard to integration into grooming networks between the period subsequent to re-socialisation and the second year of group-life in the ex-laboratory chimpanzees (Table 2 & Supplementary Table 2). In the second year, ELDs (*n* = 10) continued to have a significantly lower *vertex strength centrality* than either LLDs (*n* = 8) or NDs (*n* = 16; ELD vs. LLD: Mann-Whitney *U* = 8.0, *P* = 0.005; ELD vs. ND: *U* = 6.5, *P* < 0.001; Fig. 4a), but stopped to differ significantly from EMDs (*n* = 7; ELD vs. EMD: *U* = 12.5, *P* = 0.032). With regard to their *deviation from the edge weight disparity* ELDs (*n* = 2) and LLDs (*n* = 7) no longer differed significantly from NDs (*n* = 16; ELD vs. ND: Mann-Whitney *U* = 1.0, *P* = 0.026; LLD vs. ND: *U* = 32.0, *P* = 0.118; Fig. 4b). This can be traced back to an increased variability of the two ELDs who performed any grooming as well as the LLDs (see Fig. 4b). Overall, ELDs could not recover most of the differences within the two years after re-socialisation as can be seen in Fig. 3. Since we found similar differences in the EMDs, some of whom had been living in a stable social group for more than 40 years, this can hardly be surprising.

## Discussion

In this study, we compared chimpanzees with different deprivation histories with non-deprived zoo chimpanzees. We found that ex-laboratory chimpanzees (ELDs and LLDs) were much more restricted regarding their association partners, indicated by their higher deviation from edge weight disparity, than were non-deprived (ND) but also wild-caught orphaned (EMDs) zoo subjects. While early long-term deprived ex-laboratory chimpanzees (ELDs) also were significantly less strongly associated with their group members subsequent to re-socialisation than were EMDs and NDs, it seems that some ex-laboratory chimpanzees recovered during the second year of group-life. This is indicated by their proximity values becoming more similar to those of the zoo chimpanzees and by the fact that some LLD chimpanzees developed proximity networks comparable to those of EMDs and NDs. Thus, it seems that social stability is very important for tolerating conspecifics near-by.

In contrast to the toleration of close proximity, social grooming activity was affected by early maternal loss. The grooming activity of early long-term deprived ex-laboratory chimpanzees (ELD) and of early maternally deprived zoo chimpanzees (EMD), but not that of later deprived ex-laboratory chimpanzees (LLD) was significantly reduced in comparison with that of non-deprived zoo chimpanzees (ND). In other words, early maternally deprived chimpanzees, irrespective of whether they spent their later lives in a laboratory or in a zoo, exhibited compromised grooming networks compared to non-deprived zoo chimpanzees (ND). In the EMD zoo chimpanzees this effect was visible after decades of group living, and in the ELD ex-laboratory chimpanzees, these impaired grooming networks had not improved after two years of group-life. Social grooming, thus, seems to be affected by early maternal deprivation, even though small social group sizes during development may have caused social deficits in EMD chimpanzees as well.

Comparisons with regard to deviation from edge weight disparity of grooming networks of ELD chimpanzees have little power due to the remaining small sample size (*n* = 2). That this sample size is so small is a result in itself. Deviation from edge weight disparity could only be calculated for those individuals who actually did groom. The fact that even after two years of group-life only two out of 10 ELDs groomed at all (see also Fig. 3), serves to reinforce the statistical result found subsequent to re-socialisation.

To our knowledge, this is the first evidence for the long-term effects of early maternal loss on social competence in chimpanzees. This profound impairment of grooming activity can be traced far into adulthood, continuing to be evident in some 45 + years-old EMD chimpanzees that have been living in stable social groups for many years. Yet, while we did control for age classes^22^ in our models, we could not fully rule out that their reduced grooming activity might simply be caused by old age, because many of the EMDs were very old at the time of this study. For this reason, we compared the grooming network measures of our old EMDs with another data set for mature wild-caught orphaned chimpanzees at the Arnhem Zoo collected some 30 years earlier (between 1976 and 1985). We found no significant differences between our old EMDs and the additional data set of EMDs who were in their prime. However, also mature EMDs of the additional sample differed significantly from our NDs (see Supplementary Table 3).

An influence of early attachment experience on grooming behaviour later in life has recently been demonstrated also for captive-born ex-pet and ex-performer chimpanzees. These chimpanzees had been exposed to varying degrees of human and conspecific contact during their first four years of life. Those chimpanzees who had a high proportion of contact to conspecifics during infancy groomed more than those who did not have such an experience^42^. In this study, we found social grooming to be impaired or even lacking in those chimpanzees who were captured from the wild and experienced maternal loss in early infancy (EMDs and ELDs). Long-term deprived individuals who lost their mothers later, as juveniles, performed intermediate and did not differ from either NDs or EMDs. Taken together with the findings on human-exposed ex-pet and ex-performer chimpanzees it is highly likely that the reduced grooming activity of early maternally deprived chimpanzees can be attributed to a lack of learning. This is in line with the findings in normal wild chimpanzees, where infants’ grooming directed towards the mother increases steadily over the first years of life^41^, and in nursery and peer-reared infants where the appropriate use of grooming gestures towards social partners develops beyond the first year of life^43^.

In chimpanzee infants, social activity is highly dependent on arousal levels, which in turn are modulated by the care-giving environment, i.e., the availability of a primary attachment figure serving as a secure base^31^. Moreover, early attachment experience and affect regulation are highly interdependent in nonhuman^25^ and human primates^44^. Adult humans, for instance, reported to perceive intimacy as aversive if they experienced the avoidance of proximity by attachment figures in infancy^45^. Since ELD as well as LLD chimpanzees were single caged for decades, we assume that avoidance of social over-stimulation is the main cause for their restriction regarding the conspecifics they tolerated close-by subsequent to re-socialisation. The apparent choosiness of some LLDs, however, might also have been caused by the avoidant behaviour of their socially withdrawn ELD group members^37^. It is likely that the establishment of stable social relationships with its inherent increased predictability of group members’ behaviour will lead to a greater tolerance of close proximity. This might also explain the differences we found between the zoo chimpanzees who had been living in stable social groups for many years compared to the ex-laboratory chimpanzees who had only two years to establish relationships and who may therefore find it harder to predict each other’s behaviour. The increase in tolerating conspecifics close-by of the LLDs during the second year of group-life is in line with this assumption. Moreover, the finding that females have more tightly knit proximity networks compared to males confirms previous findings for captive chimpanzees^46^ and reveals the social propensities of female chimpanzees when they are not constrained by ecological conditions. Even so, association patterns of ELD females were obviously affected by the adverse effects of long-term deprivation.

In conclusion, we showed that early maternal loss has long-term effects on the social development of chimpanzees, even though we could not control for several possible confounding factors due to the retrospective nature of our study. We found that in non-deprived captive chimpanzees social integration is characterised by strong association networks and low selectivity of grooming partners. Social stability seems to be highly important for the toleration of passive close proximity, evident in the tightly knit proximity networks of zoo chimpanzees who had been living in stable social groups for many years, and in the increased toleration of conspecifics close-by of several ex-laboratory chimpanzees during the second year of group-life. The social grooming activity, however, was significantly lower and more restricted not only in early long-term deprived ex-laboratory chimpanzees but also in early maternally deprived zoo chimpanzees compared to that of non-deprived zoo chimpanzees, despite living in the same stable groups. Thus, our data suggest, that in chimpanzees early maternal loss affects the social grooming competence throughout lifetime. We, therefore, assume that for a chimpanzee infant being reared by its mother along with other socialisation experiences in early infancy is necessary to become a competent groomer, and think that this finding is of great importance for our understanding of chimpanzees’ development of social competence in general, and for husbandry regulations in particular.

## Methods

### Subjects and data collection

The study is based on behavioural observations of 43 adult chimpanzees (for biographic information on study subjects see Supplementary Table 1) living in five different social groups. We collected data on three social groups comprising ex-laboratory chimpanzees at the primate sanctuary in Gaenserndorf, Austria subsequent to their re-socialisation in 2003, and during their second year of group-life in 2005. There is a one-male mixed-sex group of 5 adults (MS1), a two-male mixed-sex group of 6 adults and 3 immatures (MS2), and an all-male group of 7 adults (AM). The 18 adult chimpanzees could be classified as either *early long-term deprived* (ELD, *n* = 10) or *late long-term deprived* (LLD, *n* = 8). ELDs arrived at the laboratory at an estimated age of 1 to 2 years and were solitary housed for up to 27 years (mean = 23.1 ± s.d. 3.0). LLDs arrived at the laboratory at an estimated age of 3 to 4 years, spent one more year in a peer group before being single caged for up to 16 years (mean = 15.6 ± s.d. 0.5). The chimpanzees had been part of biomedical research protocols but emerged uninfected. After their socialisation, all three groups were living in separate indoor enclosures of 130 m^2^ (MS1) or 208 m^2^ (AM and MS2). The chimpanzees were fed four times a day, and water was available ad libitum. For more detailed information on these groups see Kalcher *et al.*^35^.

Data were also collected on two stable social groups of zoo chimpanzees in The Netherlands from 2010 to 2011. The social group at Burgers’ Zoo in Arnhem (Arnhem group) was established in 1971. During the study, the group consisted of 14 adults (3 males and 11 females) and 8 immatures. 3 of the 11 adult females are founder individuals who were wild-caught in their infancy. The group at Dierenpark Amersfoort (Amersfoort group) was established in 1960. During the study, the group consisted of 11 adults (1 male, 10 females) and 4 immatures. The adult male and 5 of the 10 adult females are founder individuals who were wild-caught in their infancy. We classified 23 out of the 25 adult zoo chimpanzees as either *early maternally deprived* (EMD, *n* = 7) or *non-deprived* (ND, *n* = 16). EMDs arrived in Europe at an estimated age of 1 to 2 years and were peer reared, except for two subjects who were kept solitary for 1 year before being peer reared. Two of the founder individuals who were 3 and 5 years old upon arrival were classified as *late maternally deprived* (LMD) but excluded from statistical analysis due to small sample size^47^. NDs are zoo-born and mother reared in social groups of mixed sex and age. Both groups inhabited outdoor (Arnhem: 7,000 m^2^, Amersfoort: 475 m^2^) and indoor enclosures (Arnhem: 378 m^2^, Amersfoort: 96 m^2^), were fed several times a day, and water was available ad libitum.

Additionally, we assigned subjects to two different age classes (according to ref. 22): mature (including females 13–33 years old and males 16–33 years old), and old (including males and females more than 33 years old).

We collected data on passive close proximity (i.e., being within an arm’s reach of a conspecific) and social grooming (i.e., who is grooming whom) of all adult individuals (and only among adults) by scan sampling^48^. At the primate sanctuary in Gaenserndorf we conducted 5-minute scan sampling subsequent to re-socialisation, i.e., from October 2003 to January 2004, and during the second year of group-life, i.e., from February to July 2005. Sampling was evenly distributed over the chimpanzees’ activity period in the indoor enclosures. The mean number of scans per group was 543 (range: 485–568). At Arnhem and Amersfoort, we conducted 2–4 group scans a day and collected 442 scans between November 2010 and August 2011 at Arnhem and 204 scans between January 2011 and September 2011 at Amersfoort.

### Ethical note

This purely behavioural study was carried out in accordance with the recommendations of the US National Research Council^49^, with the Austrian Federal Act on the Protection of Animals, and with the Dutch law. The retirement process of the ex-laboratory chimpanzees at the primate sanctuary in Gaenserndorf, Austria was recommended by a board of experts, including J.A.R.A.M. van Hooff (Emeritus University of Utrecht), Mike Seres (formerly MPI Leipzig), Janet Gonder (formerly Baxter) and Joerg Eichmann (then consultant to Baxter) and conducted under the direction of Signe Preuschoft. Burgers’ Zoo in Arnhem and Dierenpark Amersfoort are members of the European Association of Zoos and Aquaria, fulfilling the legal and ethical regulations on captive animal welfare. Since the study was purely behavioural, it did not meet the definition of an animal experiment as mentioned in Article 1 of the Dutch “Experiments on Animals Act.” Accordingly, the ethics committee of Utrecht University waived the need for approval.

### Social Network Analysis & Statistics

Networks were constructed in R 2.14.0^53^ using igraph 0.5.5–3^51^ to create the graphs. R script was adapted according to McFarland *et al.*^52^. In our weighted network graphs ‘vertices’ (nodes) refer to individuals, undirected ‘edges’ of close proximity graphs to the percent of scans two individuals spent within an arm’s reach, and directed ‘edges’ of grooming graphs to the percent of scans an individual (=groomer) spent on grooming the respective partner (=groomee). We calculated two different weighted network measures^53^,^54^ per individual for close proximity and social grooming, respectively:

(a) The *vertex strength centrality* 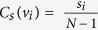 is calculated by dividing the vertex strength *s*_*i*_ by the number of group members −1 (*N* − *1*). The vertex strength *s* of vertex *i* is given by 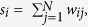 where *w* is the corresponding weight of the edges connected to a vertex. The vertex strength centrality reflects the mean percent of scans an individual spent within an arm’s reach to or grooming of an individual group member, thus showing the standardized strength of association and grooming activity, respectively, where the group size is taken into account.

(b) The *edge weight disparity* 
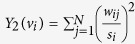
 reflects how evenly individuals distribute their association or their grooming amongst their group mates. We calculated the *deviation from edge weight disparity* to enable comparability across groups, i.e., we calculated the equal disparity Y_2_ per group which is 1/(*N* − 1) and computed the deviation from Y_2_ per individual by subtracting the group-specific Y_2_ from the individual’s Y_2_(*v*_*i*_). That means, the higher the value, the more restricted is the respective individual regarding its association or grooming partners.

In order to compare the individual weighted network measures statistically, we used Generalized Linear Mixed Models (GLMMs) in which the network measures vertex strength centrality and deviation from edge weight disparity were dependent variables and age class, sex, deprivation class (i.e., non-deprived (ND) for the mother reared zoo chimpanzees, early maternally deprived (EMD) for the early maternally deprived zoo chimpanzees, early long-term deprived (ELD) for the early maternally and socially deprived ex-laboratory chimpanzees, and late long-term deprived (LLD) for the later maternally and socially deprived ex-laboratory chimpanzees) and the 2-way interaction of sex and deprivation class were fixed factors in the full model. Group size was included as random effect. Dependent variables were normally distributed; therefore, we ran normal GLMMs with an identity link function. As we considered data structure by assigning the individuals to their respective group, we refrained from conducting permutation tests. We ran full models and reduced models based on a backward step-wise approach. Best-fitting models were chosen according to comparisons of the corrected Akaike Information Criteria (cAIC’s) (see Supplementary Table 4). Post-hoc Mann-Whitney U Tests with Holm-Bonferroni correction^55^ were performed to compare individual network measures between the different categories per predictor variable.

## Additional Information

**How to cite this article**: Kalcher-Sommersguter, E. *et al.* Early maternal loss affects social integration of chimpanzees throughout their lifetime. *Sci. Rep.* **5**, 16439; doi: 10.1038/srep16439 (2015).

## Supplementary Material

Supplementary Information

## Figures and Tables

**Table 1:** Factors influencing close proximity network measures.

	Vertex strength centrality		Deviation from edge weight disparity	
a) Subsequent to re-socialisation (2003)				
Intercept	***F***_**8,32**_** = 4.514**	***P***** = 0.001**	***F***_**3,37**_** = 12.073**	***P***** = 0.000**
Age class	*F*_1,32_ = 0.392	*P* = 0.536	—	—
Sex	***F***_**1,32**_** = 10.637**	***P***** = 0.003**	—	—
Deprivation class	***F***_**3,32**_** = 3.797**	***P***** = 0.020**	***F***_**3,37**_** = 12.073**	***P***** = 0.000**
Sex*Deprivation class	***F***_**3,32**_** = 4.317**	***P***** = 0.012**	—	—
b) Second year of group-life (2005)				
Intercept	*F*_8,32_ = 1.666	*P* = 0.146	*F*_3,36_ = 0.807	*P* = 0.498
Age class	*F*_1,32_ = 0.315	*P* = 0.578	—	—
Sex	*F*_1,32_ = 0.296	*P* = 0.590	—	—
Deprivation class	*F*_3,32_ = 1.418	*P* = 0.256	*F*_3,36_ = 0.807	*P* = 0.498
Sex*Deprivation class	*F*_3,32_ = 1.990	*P* = 0.135	—	—

Bold: *P* < 0.05. a) Generalized linear mixed model (GLMM): influence of predictors on vertex strength centrality and deviation from edge weight disparity subsequent to re-socialisation of the ex-laboratory chimpanzees. b) GLMM: influence of predictors on vertex strength centrality and deviation from edge weight disparity during the second year of group-life of the ex-laboratory chimpanzees. (Best models were chosen according to comparisons of cAIC’s).

**Table 2:** Factors influencing grooming given network measures.

	Vertex strength centrality		Deviation from edge weight disparity	
a) Subsequent to re-socialisation (2003)				
Intercept	***F***_**8,32**_** = 3.408**	***P***** = 0.006**	***F***_**6,24**_** = 10.365**	***P***** = 0.000**
Age class	*F*_1,32_ = 0.160	*P* = 0.691	—	—
Sex	*F*_1,32_ = 2.080	*P* = 0.159	***F***_**1,24**_** = 16.819**	***P***** = 0.000**
Deprivation class	***F***_**3,32**_** = 3.919**	***P***** = 0.017**	***F***_**3,24**_** = 11.953**	***P***** = 0.000**
Sex*Deprivation class	*F*_3,32_ = 2.798	*P* = 0.056	***F***_**2,24**_** = 5.213**	***P***** = 0.013**
b) Second year of group-life (2005)				
Intercept	***F***_**8,32**_** = 3.758**	***P***** = 0.003**	***F***_**6,25**_** = 4.213**	***P***** = 0.005**
Age class	*F*_1,32_ = 0.313	*P* = 0.580	—	—
Sex	*F*_1,32_ = 0.425	*P* = 0.519	***F***_**1,25**_** = 9.375**	***P***** = 0.005**
Deprivation class	***F***_**3,32**_** = 3.779**	***P***** = 0.020**	***F***_**3,25**_** = 5.236**	***P***** = 0.006**
Sex*Deprivation class	*F*_3,32_ = 2.008	*P* = 0.133	*F*_2,25_ = 2.419	*P* = 0.110

Bold: *P* < 0.05. a) Generalized linear mixed model (GLMM): influence of predictors on vertex strength centrality and deviation from edge weight disparity subsequent to re-socialisation of the ex-laboratory chimpanzees. b) GLMM: influence of predictors on vertex strength centrality and deviation from edge weight disparity during the second year of group-life of the ex-laboratory chimpanzees. (Best models were chosen according to comparisons of cAIC’s).
