# Corrigendum: Early maternal loss affects social integration of chimpanzees throughout their lifetime

**DOI:** 10.1038/srep24842

**Published:** 2016-04-29

**Authors:** Elfriede Kalcher-Sommersguter, Signe Preuschoft, Cornelia Franz-Schaider, Charlotte K. Hemelrijk, Karl Crailsheim, Jorg J. M. Massen

Scientific Reports 5: Article number: 1643910.1038/srep16439; Published online: 11102015; updated: 04292016

The Acknowledgements section in this Article is incomplete.

“We thank the primate sanctuary in Gaenserndorf, Austria, and Burgers’ Zoo, Arnhem, and Dierenpark Amersfoort, The Netherlands, and especially all the keepers for their great enthusiasm and cooperation during the study. We also would like to thank Sabine Macherhammer, Christine Hrubesch, Alexandra Antonides and Anne-Marie Arnold for help with data collection, Claudia Kasper for valuable advice, Tom de Jongh and Raymond van der Meer for providing information about founder chimpanzees, and the Land Steiermark (A28163500023) and the Lucy Burgers Foundation for Comparative Behaviour Research for financial support.”

should read:

“We thank the primate sanctuary in Gaenserndorf, Austria, and Burgers’ Zoo, Arnhem, and Dierenpark Amersfoort, The Netherlands, and especially all the keepers for their great enthusiasm and cooperation during the study. We also would like to thank Sabine Macherhammer, Christine Hrubesch, Alexandra Antonides and Anne-Marie Arnold for help with data collection, Claudia Kasper for valuable advice, Tom de Jongh and Raymond van der Meer for providing information about founder chimpanzees. Finally, we thank the Land Steiermark (A28163500023) for financial support to C.F.-S. and the Lucy Burgers Foundation for Comparative Behaviour Research, and the Austrian Science Fund (FWF: project P26806-B22) for financial support to J.J.M.M.”

