# Supplementary material for: Early maternal loss affects social integration of chimpanzees throughout their lifetime
Source: Sci Rep. 2015 Nov 10;5:16439. doi: 10.1038/srep16439 (PMC4639738; doi:10.1038/srep16439)
Supplement: Supplementary Information [file srep16439-s1.pdf]

Supplementary Information

Early maternal loss affects social integration of chimpanzees throughout their lifetime

Elfriede Kalcher-Sommersguter, Signe Preuschoft, Cornelia Franz-Schaider, Charlotte K. Hemelrijk, Karl Crailsheim & Jorg J. M. Massen

Supplementary Table 1. Biographic information on the subjects

Name	Sex	Est. Age at Onset of Deprivation	Years Spent Single Housed	Age at Onset of Observation	Deprivation Class
		**ALL–MALE GROUP (EX–LAB)**			
Gogo	M	2	27	29	ELD
Max	M	1	24	25	ELD
Isidor	M	1	24	25	ELD
Johannes	M	1	21	22	ELD
Michi	M	1	21	22	ELD
Blacky	M	1	17	18	ELD
Jakob	M	3	16	20	LLD
		**MIXED–SEX GROUP 1 (EX–LAB)**			
Clyde	M	4	16	21	LLD
Pünktchen	F	3	16	20	LLD
Martha	F	1	24	25	ELD
Ingrid	F	1	23	24	ELD
Gabi	F	1	23	24	ELD
		**MIXED–SEX GROUP 2 (EX–LAB)**			
Moritz	M	3	16	20	LLD
Anton	M	4	16	21	LLD
Schuscha	F	4	15	21	LLD
Helene	F	4	15	21	LLD
Bonnie	F	4	15	21	LLD
Susi	F	2	27	29	ELD
		**ARNHEM GROUP (ZOO)**			
Fons	M	-	-	35	ND
Gaby	F	-	-	26	ND
Giambo	M	-	-	21	ND
Geisha	F	-	-	17	ND
Mama	F	1	0	53	EMD
Moniek	F	-	-	33	ND
Morami	F	-	-	23	ND
Marka	F	-	-	26	ND
Tepel	F	5	0	51	LMD*
Teshua	F	-	-	24	ND
Tushi	F	-	-	18	ND
Jimmy	F	1	1[b]	50	EMD

Jing	M	-	-	29	ND
Roos[a]	F	-	-	31	ND
		AMERSFOORT GROUP (ZOO)			
Mike	M	2	1[c]	45	EMD
Sonja	F	2	0	46	EMD
Belle	F	-	-	33	ND
Willy	F	-	-	19	ND
Sjors	F	1	0	44	EMD
Kokkie	F	2	0	44	EMD
Jet	F	3	3[b]	40	LMD*
Silvia	F	2	0	36	EMD
Sanne	F	-	-	21	ND
Bibi	F	-	-	14	ND
Chura	F	-	-	13	ND

Notes: Estimated age of maternally deprived zoo chimpanzees is based on Carlsen[44]. Tom de Jongh (EEP vice-coordinator) and Raymond van der Meer (curator Dierenpark Amersfoort) provided information regarding early rearing conditions of founder chimpanzees.
[a]adopted and reared by Mama, [b]Private Owner, [c]Circus; ELD = early and long-term deprived, LLD = late and long-term deprived, EMD = early maternally (and short-term) deprived, LMD = late maternally (and short-term) deprived, ND = non-deprived.
*Not considered in statistical analysis due to small sample size.

Supplementary Table 2. Medians (and range) of vertex strength centrality and deviation from edge weight disparity of close proximity and grooming given, respectively, for the different predictors

**Dependent Variable**	**Predictor**<br>Sample Size, Median, Range	**Mann-Whitney U**<br>w/ Holm-Bonferroni

CLOSE PROXIMITY

Subsequent to Re-socialisation

Age Class

Mature	Old	Mann-Whitney U
VSC: *n*=33, 1.9, 0.2-7.4<br>DEWD: *n*=33, 0.08, 0.01-0.80	VSC: *n*=8, 3.3, 1.2-4.1<br>DEWD: *n*=8, 0.02, 0.01-0.11	

Sex

Male	Female	Mann-Whitney U
VSC: *n*=14, 1.2, 0.2-4.7<br>DEWD: *n*=14, 0.13, 0.02-0.34	VSC: *n*=27, 3.8, 0.2-7.4<br>DEWD: *n*=27, 0.04, 0.01-0.80	VSC: ***U*=65.0, *P*=0.001**

Deprivation Class

ELD	LLD	EMD	ND	Mann-Whitney U
VSC: *n*=10, 1.2, 0.2-1.6<br>DEWD: *n*=10, 0.20, 0.05-0.80	VSC: *n*=8, 1.5, 0.2-7.4<br>DEWD: *n*=8, 0.20, 0.07-0.31	VSC: *n*=7, 3.4, 1.2-4.1<br>DEWD: *n*=7, 0.02, 0.01-0.11	VSC: *n*=16, 4.1, 1.9-5.7<br>DEWD: *n*=16, 0.02, 0.01-0.10	VSC: **ELD vs. EMD: *U*=4.0, *P*=0.001, ELD vs. ND: *U*=0.0, *P*<0.001**<br>DEWD: **ELD vs. EMD: *U*=3.0, *P*=0.001, ELD vs. ND: *U*=3.0, *P*<0.001, LLD vs. EMD: *U*=1.0, *P*=0.001, LLD vs. ND: *U*=1.0, *P*<0.001**

Sex*Deprivation Class

Male				Female				Mann-Whitney U
ELD	LLD	EMD	ND	ELD	LLD	EMD	ND	
VSC: *n*=6, 0.8, 0.4-1.5<br>DEWD: *n*=6, 0.18, 0.05-0.34	VSC: *n*=4, 1.0, 0.2-1.8<br>DEWD: *n*=4, 0.23, 0.07-0.31	VSC: *n*=1, 1.2<br>DEWD: *n*=1, 0.02	VSC: *n*=3, 3.1, 3.1-4.7<br>DEWD: *n*=3, 0.02, 0.02-0.02	VSC: *n*=4, 1.3, 0.2-1.6<br>DEWD: *n*=4, 0.26, 0.09-0.80	VSC: *n*=4, 5.2, 1.2-7.4<br>DEWD: *n*=4, 0.20, 0.17-0.31	VSC: *n*=6, 3.6, 2.5-4.1<br>DEWD: *n*=6, 0.03, 0.01-0.11	VSC: *n*=13, 4.1, 1.9-5.7<br>DEWD: *n*=13, 0.02, 0.01-0.10	VSC: **ELD_f vs. EMD_f: *U*=0.0, *P*=0.010, ELD_f vs. ND_f: *U*=0.0, *P*=0.001**

Supplementary Table 2 cont.

2nd Year of Group-life

Age Class							
Mature				Old			
VSC: n=33, 3.5, 0.0-5.9<br>DEWD: n=32, 0.09, 0.01-0.83				VSC: n=8, 3.3, 1.2-4.1<br>DEWD: n=8, 0.02, 0.01-0.11			
**Sex**							
Male				Female			
VSC: n=14, 1.8, 0.0-4.7<br>DEWD: n=13, 0.17, 0.02-0.83				VSC: n=27, 3.8, 0.2-5.9<br>DEWD: n=27, 0.04, 0.01-0.36			
**Deprivation Class**							
ELD		LLD		EMD		ND	
VSC: n=10, 0.3, 0.0-3.6<br>DEWD: n=9, 0.36, 0.10-0.83		VSC: n=8, 4.3, 0.1-5.9<br>DEWD: n=8, 0.15, 0.04-0.83		VSC: n=7, 3.4, 1.2-4.1<br>DEWD: n=7, 0.02, 0.01-0.11		VSC: n=16, 4.1, 1.9-5.7<br>DEWD: n=16, 0.02, 0.01-0.10	
**Sex*Deprivation Class**							
Male				Female			
ELD	LLD	EMD	ND	ELD	LLD	EMD	ND
VSC: n=6,<br>0.7, 0.0-3.6<br>DEWD: n=5,<br>0.83, 0.33-0.83	VSC: n=4,<br>2.4, 0.1-4.6<br>DEWD: n=4,<br>0.16, 0.04-0.83	VSC: n=1,<br>1.2<br>DEWD: n=1,<br>0.02	VSC: n=3,<br>3.1,3.1-4.7<br>DEWD: n=3,<br>0.02, 0.02-0.02	VSC: n=4,<br>0.3, 0.2-0.6<br>DEWD: n=4,<br>0.21, 0.10-0.36	VSC: n=4,<br>4.8, 0.3-5.9<br>DEWD: n=4,<br>0.13, 0.09-0.32	VSC: n=6,<br>3.6, 2.5-4.1<br>DEWD: n=6,<br>0.03, 0.01-0.11:	VSC: n=13,<br>4.1,1.9-5.7<br>DEWD: n=13,<br>0.02, 0.01-0.10

Supplementary Table 2 cont.

**Dependent Variable**	**Predictor**<br>Sample Size, Median, Range		**Mann-Whitney U**<br>w/ Holm-Bonferroni
GROOMING GIVEN			
Subsequent to Re-socialisation	**Age Class**		
	Mature	Old	
	VSC: *n*=33, 0.5, 0.0-4.4<br>DEWD: *n*=23, 0.18, 0.05-0.83	VSC: *n*=8, 0.2, 0.1-0.6<br>DEWD: *n*=8, 0.35, 0.08-0.90	
	**Sex**		
	Male	Female	
	VSC: *n*=14, 0.1, 0.0-1.1<br>DEWD: *n*=8, 0.75, 0.10-0.90	VSC: *n*=27, 0.6, 0.0-4.4<br>DEWD: *n*=23, 0.18, 0.05-0.90	DEWD: *U*=50.5, *P*=0.064

	**Deprivation Class**				
	ELD	LLD	EMD	ND	
Subsequent to Re-socialisation	VSC: *n*=10, 0.0, 0.0-0.1<br>DEWD: *n*=2, 0.83, 0.83-0.84	VSC: *n*=8, 0.7, 0.0-4.4<br>DEWD: *n*=6, 0.34, 0.23-0.80	VSC: *n*=7, 0.1, 0.1-0.6<br>DEWD: *n*=7, 0.40, 0.08-0.90	VSC: *n*=16, 1.1, 0.1-1.5<br>DEWD: *n*=16, 0.13, 0.05-0.35	VSC: **ELD vs. LLD: *U*=12.0, *P*=0.014,**<br>**ELD vs. EMD: *U*=4.0, *P*=0.003,**<br>**ELD vs. ND: *U*=1.0, *P*<0.001,**<br>**EMD vs. ND: *U*=8.5, *P*=0.002**<br>DEWD: **ELD vs. ND: *U*=0.0, *P*=0.013,**<br>**ELD vs. ND: *U*=6.0, *P*=0.001,**<br>**EMD vs. ND: *U*=18.0, *P*=0.010**

	**Sex*Deprivation Class**								
	Male				Female				
	ELD	LLD	EMD	ND	ELD	LLD	EMD	ND	
Subsequent to Re-socialisation	VSC: *n*=6,<br>0.0, 0.0-0.1<br>DEWD: *n*=6,<br>0.83, 0.83-0.84	VSC: *n*=4,<br>0.3, 0.0-1.1<br>DEWD: *n*=4,<br>0.75, 0.69-0.80	VSC: *n*=1,<br>0.1<br>DEWD: *n*=1,<br>0.90	VSC: *n*=3,<br>0.7, 0.6-1.1<br>DEWD: *n*=3,<br>0.11, 0.10-0.19	VSC: *n*=4,<br>0.0, 0.0-0.0<br>DEWD: *n*=0	VSC: *n*=4,<br>1.5, 0.4-4.4<br>DEWD: *n*=4,<br>0.31, 0.23-0.35	VSC: *n*=6,<br>0.2, 0.1-0.6<br>DEWD: *n*=6,<br>0.35, 0.08-0.90	VSC: *n*=13,<br>1.1, 0.1-1.5<br>DEWD: *n*=13,<br>0.14, 0.05-0.35	DEWD: LLD_f vs. ND_f: *U*=6.0, *P*=0.023,<br>EMD_f vs. ND_f: *U*=15.0, *P*=0.037

Supplementary Table 2 cont.

2nd Year of Group-life	**Age Class**								
	Mature				Old				
	VSC: *n*=33, 0.7, 0.0-2.9<br>DEWD: *n*=24, 0.17, 0.05-0.83				VSC: *n*=8, 0.2, 0.1-0.6<br>DEWD: *n*=8, 0.35, 0.08-0.90				
	**Sex**								
	Male				Female				
	VSC: *n*=14, 0.3, 0.0-1.6<br>DEWD: *n*=9, 0.35, 0.10-0.90				VSC: *n*=27, 0.7, 0.0-2.9<br>DEWD: *n*=23, 0.17, 0.05-0.90				DEWD: ***U*=55.5, *P*=0.047**
	**Deprivation Class**								
	ELD		LLD		EMD		ND		
	VSC: *n*=10, 0.0, 0.0-0.6<br>DEWD: *n*=2, 0.59, 0.35-0.83		VSC: *n*=8, 0.8, 0.0-2.9<br>DEWD: *n*=7, 0.28, 0.06-0.83		VSC: *n*=7, 0.1, 0.1-0.6<br>DEWD: *n*=7, 0.40, 0.08-0.90		VSC: *n*=16, 1.1, 0.1-1.5<br>DEWD: *n*=16, 0.13, 0.05-0.35		†<br>VSC: **ELD vs. LLD: *U*=8.0, *P*=0.005, ELD vs. ND: *U*=6.5, *P*<0.001**
	**Sex*Deprivation Class**								
	Male				Female				
	ELD	LLD	EMD	ND	ELD	LLD	EMD	ND	
	VSC: *n*=6,<br>0.0, 0.0-0.6<br>DEWD: *n*=2,<br>0.59, 0.35-0.83	VSC: *n*=4,<br>0.5, 0.0-1.6<br>DEWD: *n*=3,<br>0.63, 0.28-0.83	VSC: *n*=1,<br>0.1<br>DEWD: *n*=1,<br>0.90	VSC: *n*=3,<br>0.7, 0.6-1.1<br>DEWD: *n*=3,<br>0.11, 0.10-0.19	VSC: *n*=4,<br>0.0, 0.0-0.0<br>DEWD: *n*=0	VSC: *n*=4,<br>1.2, 0.8-2.9<br>DEWD: *n*=4,<br>0.17, 0.06-0.52	VSC: *n*=6,<br>0.2, 0.1-0.6<br>DEWD: *n*=6,<br>0.40, 0.08-0.90	VSC: *n*=13,<br>1.1, 0.1-1.5<br>DEWD: *n*=13,<br>0.14, 0.05-0.35	

Abbr.: ELD = early and long-term deprived, LLD = late and long-term deprived, EMD = early maternally (and short-term) deprived, ND = non-deprived, VSC = vertex strength centrality, DEWD = deviation from edge weight disparity.

Significant outcomes of post-hoc Mann-Whitney U tests after Holm-Bonferroni correction are shown in bold.

† Note that values for the zoo chimpanzees did not change and thus for comparisons between EMD and ND we refer to the earlier analyses (subsequent to re-socialisation) above.

Supplementary Table 3. Medians (and range) of vertex strength centrality and deviation from edge weight disparity of grooming given for *mature* early maternally deprived (EMD$_M$), *old* early maternally deprived (EMD$_O$) and non-deprived (ND) chimpanzees in comparison

**Dependent Variable**	**Predictor** Sample Size, Median, Range			**Mann-Whitney U** w/ Holm-Bonferroni
GROOMING GIVEN				
	**Age Class**			
	EMD$_M$*	EMD$_O$	ND	
	VSC: *n*=7, 0.1, 0.0-0.5<br>DEWD: *n*=7, 0.33, 0.12-0.50	VSC: *n*=7, 0.1, 0.1-0.6<br>DEWD: *n*=7, 0.40, 0.08-0.90	VSC: *n*=16, 1.1, 0.1-1.5<br>DEWD: *n*=16, 0.13, 0.05-0.35	VSC: EMD$_M$ vs. EMD$_o$: U = 22.0, P=0.805,<br>**EMD$_M$ vs. ND: U=7.0, P<0.001,**<br>**EMD$_O$ vs. ND: U=8.5, P=0.002**<br>DEWD: EMD$_M$ vs. EMD$_o$: U = 17.0, P=0.383,<br>**EMD$_M$ vs. ND: U=17.0, P=0.008,**<br>**EMD$_O$ vs. ND: U=18.0, P=0.010**

Note: *EMD$_M$ comprises seven adult females (for details see Table below) from Arnhem zoo. Vertex strength centrality and deviation from edge weight disparity were calculated out of individual medians for every groomer-groomee dyad over a 10-year period (1976-1985). Charlotte Hemelrijk provided scan data.

**Name**	**Est. Age at Onset of Deprivation[1]**	**Age in 1976[3]**	**Observation period**	**Number of Available Adult Grooming Partners 1976-1985**
Mama	1	20	1976-1985	7-15
Puist	?	16	1976-1985	7-15
Gorilla	2[2]	19	1976-1985	7-15
Jimmy	1	16	1976-1985	7-15
Franje	2[2]	19	1976-1979	7-9
Krom	1	15	1976-1984	7-14
Spin	?	16	1976-1985	7-15

[1]Carlsen, F. European studbook for the chimpanzee *Pan troglodytes* (Copenhagen Zoo, 2009).
[2]Based on information and a picture provided by Tom de Jongh (EEP vice-coordinator).
[3]Adang, O. M. J., Wensing, J. A. B. & van Hooff, J. A. R. A. M. The Arnhem Zoo colony of chimpanzees *Pan troglodytes*: development and management techniques. *Int. Zoo Yb*. **26**, 236-248 (1987).

Supplementary Table 4. Model selection according to comparisons of the corrected Akaike Information Criteria (cAICs)

	Dependent Variable	Model	F	P	cAIC	Δ cAIC
**CLOSE PROXIMITY a) subsequent to re-socialisation for ex-laboratory chimpanzees**	Vertex strength centrality	**Full Model**	**4.514**	**0.001**	**112.444**	
		Full Model - Age class	5.176	0.000	115.144	-2.700
	Deviation from edge weight disparity	Full Model	3.662	0.004	-32.485	
		Reduction 1: Full Model - Age class	4.040	0.003	-34.629	2.144
		Reduction 2: Reduction 1 - Sex*Deprivation class	9.689	0.000	-40.034	5.405
		**Reduction 3: Reduction 2 - Sex**	**12.073**	**0.000**	**-42.859**	**2.825**
**CLOSE PROXIMITY b) 2nd of group-life for ex-laboratory chimpanzees**	Vertex strength centrality	Full Model	1.666	n.s.		
	Deviation from edge weight disparity	Full Model	0.807	n.s.		
**GROOMING GIVEN a) subsequent to re-socialisation for ex-laboratory chimpanzees**	Vertex strength centrality	**Full Model**	**3.408**	**0.006**	**76.720**	
		Full Model - Age class	3.956	0.003	78.114	-1.394
		Full Model - Sex	3.408	0.006	76.720	0.000
		Full Model - Sex*Deprivation class	3.328	0.015	87.614	-10.894
	Deviation from edge weight disparity	Full Model	8.623	0.000	-9.405	
		**Full Model - Age class**	**10.365**	**0.000**	**-10.972**	**1.567**
**GROOMING GIVEN b) 2nd of group-life for ex-laboratory chimpanzees**	Vertex strength centrality	**Full Model**	**3.758**	**0.003**	**57.115**	
		Full Model - Age class	4.338	0.002	57.998	-0.883
		Full Model - Sex	3.758	0.003	57.115	-0.000
		Full Model - Sex*Deprivation class	4.473	0.003	64.130	-7.015
	Deviation from edge weight disparity	Full Model	3.484	0.100	0.863	
		**Reduction 1: Full Model - Age class**	**4.213**	**0.005**	**-0.368**	**1.231**
		Reduction 2: Reduction 1 - Sex*Deprivation class	4.618	0.006	1.417	-1.785

Bold: Best-fitting models according to comparisons of cAICs; if Δ cAIC > 2, we chose the new model (Burnham and Anderson 2002), and if 0 < Δ cAIC < 2, we chose the most parsimonious model.

Supplemental references:

Burnham, K. P. & Anderson, D. R. *Model selection and multimodel inference: a practical information-theoretic approach* (Springer, 2002).
